# The *Nicotiana benthamiana* Biofactory

**DOI:** 10.1111/pbi.70547

**Published:** 2026-01-14

**Authors:** Dominique Michaud, Stephen J. Streatfield

**Affiliations:** ^1^ Département de phytologie, Centre de recherche et d'innovation sur les végétaux Université Laval Québec QC Canada; ^2^ Fraunhofer USA Center for Molecular Biotechnology Newark DE USA

The idea of using plants as biofactories to produce medically valuable proteins was formalized some 35 years ago by Andy Hiatt, Robert Cafferkey and Katherine Bowdish in a paper reporting the successful expression of mammalian antibodies in tobacco (Hiatt et al. 1989), soon followed by Peter Sijmons and co‐workers describing the correct processing of human serum albumin in potato (Sijmons et al. 1990). These seminal papers, followed by thousands of others over the following decades, have paved the way for the emergence of ‘plant molecular farming’, a now thriving discipline of plant biotechnology dedicated to the heterologous production of valuable proteins and organics in plant systems. Plant‐based expression platforms have been developed to produce a wide array of valuable recombinant products, including vaccine antigens, therapeutic antibodies, bioactive proteins and (poly)peptides (Chaudhary et al. 2024; Eidenberger et al. 2023; Stander et al. 2022), and, more recently, small organic chemicals (Golubova et al. 2024; Liu et al. 2023). The commercialization of plant‐made recombinant protein products and the expected approval of several others for human use in the coming years have strengthened the position of plant molecular farming as a mature, viable option for the heterologous production of useful protein and organic products (Washida et al. 2025; Anon. 2022).

One reason for the success of plant molecular farming is the development of transient expression platforms involving leaf infiltration with engineered *Agrobacterium tumefaciens* (now referred to as *Rhizobium radiobacter* ) (Akher et al. 2025) harboring expression vectors optimized for plant‐based protein production. Originally used to quickly screen for functional promoter sequences and gene constructs, transient expression in agroinfiltrated leaves has since developed into the fastest and most convenient production platform for plant‐made (bio)pharmaceuticals. This approach relies on the ability of the *A. tumefaciens* Ti plasmid to transfer a transcriptionally competent segment of DNA into the plant's host cell, that is then directed to the nucleus for heterologous expression. Several transient expression systems have been devised over the years, that enable the production of milligram quantities of recombinant proteins within a few days in a handful of plants.

Most of these systems make use of the wild tobacco relative *Nicotiana benthamiana* as an expression host (Bally et al. 2018). Widely adopted as an experimental model to elucidate plant‐pathogen interactions, this plant rapidly generates leaf biomass and is easily amenable to agroinfiltration for transgene introduction and high‐level expression. In recent years, *N. benthamiana* has become the most widely used host for transient protein production and several research papers are published every week, if not daily, reporting on the successful expression of a valuable recombinant protein in this plant or the reconstruction of a metabolic pathway to produce a useful metabolite. Building on the increasing importance of *N. benthamiana* in plant biotechnology and the instrumental role played by *PBJ* in the development of molecular farming platforms over the last 20 years, this special issue provides a collection of state‐of‐the‐art articles at the forefront of current developments in the field. Authoritative, incisive reviews first address specific topics pertaining to the *N. benthamiana* expression platform, from the host plant's response to agrobacterial infection and current strategies for transgene expression to recombinant protein design, environmental control and downstream processing of the recombinant protein product. Top‐notch primary research papers then present original data reporting on the production of a new biopharmaceutical, proposing a new approach to improve recombinant protein yield and/or quality in leaf tissue, or describing a unique metabolic engineering strategy to produce an organic compound of clinical interest.

The current issue includes 28 papers, including 6 reviews, 6 brief communications and 16 full‐length research articles. A complementary virtual issue will also be published on the journal website, including the same 28 papers along with relevant papers published in regular issues in 2025 and a few more papers, including an additional review, still in the production pipeline. Once completed, the special issue –as available via the virtual issue– will include around 40 high‐quality contributions from nearly 40 laboratories all over the world. We would like to express our thanks and appreciation to all contributors for their active participation to this collective work, which arguably represents one of the most, if not the most, comprehensive publication effort yet entirely dedicated to the *N. benthamiana* biofactory. We also wish to express our deepest gratitude to the more than 100 external reviewers recruited along the way, who by their expertise and hard work directly contributed to the overall quality of the resulting outcome. Last but not the least, we thank the Wiley team for their precious assistance during the project, and the Editor‐in‐Chief of PBJ, Prof. Johnathan Napier, for his valuable suggestions and continued support from the start to the end.

Dominique Michaud, Senior Editor, PBJ.

Stephen J. Streatfield, Associate Editor, PBJ.

## Data Availability

Data sharing not applicable to this article as no datasets were generated or analyzed during the current study.
